# Trends and challenges in computational RNA biology

**DOI:** 10.1186/s13059-016-1117-7

**Published:** 2016-12-07

**Authors:** Alina Selega, Guido Sanguinetti

**Affiliations:** School of Informatics, University of Edinburgh, Edinburgh, UK

**Keywords:** RNA, Review, Computational biology

## Abstract

A report on the Wellcome Trust Conference on Computational RNA Biology, held in Hinxton, UK, on 17–19 October 2016.

## Introduction

Recent years have witnessed a profound shift in our understanding of RNA biology. Several novel biochemical and sequencing techniques are producing vast amounts of data that fundamentally challenge the textbook view of RNA as a simple intermediate step of gene expression, revealing a wealth of unexpected new roles and shedding light on the complexity of the RNA world. While the emerging picture unequivocally points to the centrality of RNA as a mediator of most cellular functions, the richness and heterogeneity of modern datasets pose significant interpretative challenges and call for an interdisciplinary approach where statistical and computational methods will play an increasingly important role.

The Wellcome Trust Conference on Computational RNA Biology provided a good opportunity to overview the state of the art in this up-and-coming interdisciplinary field. Organised by the scientific committee of Alex Bateman (European Bioinformatics Institute, UK), Ivo Hofacker (University of Vienna, Austria), Karissa Sanbonmatsu (Los Alamos National Labs, USA) and Mihaela Zavolan (University of Basel, Switzerland), the conference was held at the Wellcome Genome Campus in Hinxton, near Cambridge (UK) on 17–19 October 2016. Featuring two keynote talks by Christine Mayr (Memorial Sloan Kettering Cancer Center, New York, USA) and Ben Blencowe (University of Toronto, Canada), thirteen invited talks and fourteen short contributed talks, the conference provided a very broad survey of quantitative and computational RNA biology. These were further complemented by two lively poster sessions, where participants had an opportunity to engage with over 40 posters during evening drinks receptions.

In this report, we briefly recount the content of the conference by providing condensed, headline-style summaries of the research described in the talks and some posters. Within the scope of this brief report, we cannot possibly do justice to the wealth and breadth of material presented and we will not be able to mention much interesting research, particularly within the poster sessions. We would like to stress that omissions in this report are not based on quality, but simply on a personal judgement as to what material could be most coherently presented in a very limited space.

## Transcripts

Perhaps the most remarkable discovery in modern RNA biology is the realization of the diversity of the transcriptome. Technologies based on next-generation sequencing (NGS) have demonstrated the existence of many novel classes of transcripts and the great variety of protein-coding transcripts, in terms of both isoforms and synonymous variants. The diversity of the transcriptome and its interaction with phenotypes was the main theme of both keynote talks. Ben Blencowe (University of Toronto) introduced the concept of alternative splicing regulatory networks and their role in development and autistic spectrum disorders. Blencowe illustrated how analysis of NGS data has enabled the discovery of a novel class of micro-exons (3–27 nucleotides) that are strongly conserved and whose alternative exclusion is associated with the autism phenotype. Isoform quantification methods were discussed by Eduardo Eyras (University of Pompeu Fabra, Barcelona, Spain), who explained how the SUPPA method achieves high computational performance by decoupling read mapping from transcript annotation. Methodologies for isoform quantification from time series RNA-seq data using the DICEseq method were also presented in the poster session by Yuanhua Huang (University of Edinburgh, UK). Naturally, the presence of isoform RNA molecules does not immediately imply isoform expression at the protein level, as translational regulation may preferentially select only a subset of isoforms. This question was addressed by Lorenzo Calviello (Max Delbrück Center for Molecular Medicine, Berlin, Germany), who used ribosome profiling data and the Splice-aware Translational Annotation (SaTAnn) tool. This analysis revealed that almost 55% of genes (in human HEK293 cells) translate a single isoform, and highlighted widespread translational control. SaTAnn also received the Best Acronym Award, beating stiff competition from CRAC and BUM-HMM (see below).

While alternative splicing has long been recognized as a major determinant of the diversity of the transcriptome, recent research is also shedding light on the functional significance of synonymous variants, i.e. transcripts that differ only in the non-coding region. Christine Mayr (Memorial Sloan Kettering Cancer Center, New York) described how transcript variants with different 3^′^ UTRs can give rise to dramatically different functions in the protein they code for. A prominent example is given by the CD47 transcript in human: variants with a long 3^′^ UTR are preferentially bound by the HuR protein (due to the abundance of HuR binding sites on the long UTR), which then leads to membrane localization of the nascent protein, while CD47 proteins synthesized from a short 3^′^ UTR variant remain in a perinuclear localization. Shorter transcript variants can also arise from alternative use of polyA sites, the presentation topic of Christina Leslie (also from Memorial Sloan Kettering Cancer Center, New York), although in this case the shorter transcript mostly results in a truncated protein or in a non-coding RNA (ncRNA).

The discovery of a great variety of novel ncRNAs was also one of the major breakthroughs of NGS technologies; ncRNAs remain, however, largely mysterious in their biological function. Albin Sandelin (Biotech Research & Innovation Centre, Copenhagen, Denmark) described data from cap analysis of gene expression (CAGE) experiments illustrating the pervasiveness of bidirectional transcription, often giving rise to mRNA–ncRNA pairs. He further explained how genomic features such as density of polyA sites or closely spaced transcription start sites influence ncRNA expression. Igor Ulitsky (Weizmann Institute, Rehovot, Israel) used synteny to elucidate the function and origin of lincRNAs (long intergenic ncRNAs), highlighting a modest level of sequence conservation, partly explained by the presence of enhancers within lincRNAs. Sequence comparison methodologies, initially developed to study paralog genes, were also discussed by Jana Hertel (University of Leipzig, Germany) to address ncRNA evolution. Finally, Todd Lowe (University of California, Santa Cruz, USA) used chromatin data from the Encyclopedia Of DNA Elements (ENCODE) project to discover widespread epigenetic regulation of the human tRNA transcriptome.

## Structures

RNAs in vivo fold in complex secondary and 3D structures. It is widely believed that RNA structures play a major regulatory role in determining the possible interaction partners of RNAs, and ultimately, their function. The computational biology community has long had a sustained interest in predicting RNA structures and the conference witnessed several interesting presentations on the matter.

While in principle feasible configurations could be computed by minimizing free energies derived from microscopic physical principles, the computation is in general prohibitively complex. Simon Poblete (International School for Advanced Studies, Trieste, Italy) presented a novel approach to coarse-graining the state space of possible configurations, leading to considerable accelerations in molecular dynamics simulations. Other talks described approaches that instead use auxiliary data to bypass the difficult step of molecular simulations. Craig Zirbel (Bowling Green State University, USA) described JAR3D, a set of probabilistic models parametrized on the RNA 3D Motif Atlas, that infer new 3D motifs from sequences. Debora Marks (Harvard Medical School, USA) described how evolutionary couplings can be used within global probability models to improve the predictive power of optimisation algorithms. Evolutionary arguments can also be invoked to exploit pairwise covariations in multiple RNA alignments to deduce the conservation of RNA secondary structures. This line of reasoning was used by Elena Rivas (Harvard University, USA) to argue against the conservation of secondary structures in long ncRNAs, stirring a certain level of debate within the conference. Mutation patterns underlying structure conservation were also employed by Zasha Weinberg (University of Leipzig) to discover a new group of riboswitches (metabolite-binding RNAs) and by Martin Smith (Garvan Institute, Sydney, Australia) to cluster evolutionarily conserved RNA structural patterns.

A major source of excitement within the RNA structure community is the development of novel sequencing-based techniques for structure probing in vivo. High-throughput experiments using a variety of probing agents are being performed at an increasing pace and Yiliang Ding (John Innes Centre, Norwich, UK) described FoldAtlas, a curated repository for such data that is likely to become a precious resource. In poster sessions, Krishna Choudhary (University of California, Davis, USA) highlighted the importance of quality control by presenting metrics for rapid quality assessment of structure probing data. Alain Laederach (University of North Carolina at Chapel Hill, USA) showed how structure probing techniques led to the discovery of riboSNitches, mutations in the non-coding part of a transcript which can alter the secondary structure of the UTR, leading to functional changes with often dramatic associations with disease phenotypes. Alina Selega (University of Edinburgh) described BUM-HMM, a novel probabilistic model for controlling for biological variability within high-throughput structure probing data. Mirko Ledda (University of California, Davis) presented a probabilistic model to incorporate structure probing data into a pseudo-free energy term used in folding prediction algorithms.

## Interactions

Another major focus of the conference was the discussion of the RNA interactome. Indeed, many of the exciting discoveries in recent RNA biology are due to the exceptional flexibility of RNA as an interacting molecule, acting on DNA, other RNAs and proteins. Characterizing these interactions quantitatively is a primary avenue of research in RNA biology, both experimentally and computationally.

Protein–RNA interactions are primarily identified via cross-linking with ultraviolet (UV) light using the cross-linking and immunoprecipitation (CLIP) protocol. Rolf Backofen (University of Freiburg, Germany) described GraphProt, a computational approach to detect RNA–protein binding motifs from CLIP data in a supervised learning pipeline using sequence and (predicted) structural features. Andre Gerber (University of Surrey, UK) used cross-linking to determine the entire mRNA-interacting proteome in yeast and *Caenorhabditis elegans*. He identified a large number of proteins (>600 in both species) with a very high degree of conservation. Remarkably, a large fraction of RNA-binding proteins (RBPs) turned out to be metabolic enzymes interacting with RNAs belonging to the same metabolic pathways. Guido Sanguinetti (University of Edinburgh) discussed computational models of cross-linking and cDNA (CRAC) data (another UV cross-linking technique), which was used to model the changes in polymerase post-translational modifications during transcription, and the fast kinetics of co-transcriptional degradation during stress induction. Bojan Zagrovic (University of Vienna) presented data supporting the hypothesis that translation originated by direct interactions between codons and amino acids, an old idea from Carl Woese that is now being tested with modern technologies.

RNA–RNA interactions also play a central role in many regulatory processes; Yair Gatt (Hebrew University, Jerusalem, Israel) described RIL-seq, a modification of the cross-linking, ligation and sequencing of hybrids (CLASH) method, to identify targets of small RNAs in *Escherichia coli* by measuring interactions of sRNAs bound to the Hfq protein, enabling the detection of several hundred new RNA–RNA interactions. Paul Gardner (Canterbury University, Christchurch, New Zealand) discussed new results indicating that mRNA sequences appear to be under selection in order to avoid random interactions with ncRNAs. Surprisingly, avoidance of ncRNAs seems to be a better predictor of protein abundance than codon usage.

## New technologies and applications

A number of talks in the workshop reported the development of new methodologies for both novel experiment types and technological applications. Martin Jansson (University of Copenhagen, Denmark) described how RiboMeth-seq enables measurement of 2^′^-O-methylation, a common epi-transcriptomic modification that may contribute to ribosome diversity, eventually tuning translation. Jorg Morf (Babraham Institute, Cambridge, UK) described a new bead-based method for assaying RNA–RNA proximity, yielding highly reproducible results. Fabian Amman (University of Vienna) presented a computational approach to optimize cell-free translation in synthetic biology applications. In poster sessions, Qi Liu (John Innes Centre, Norwich, UK) demonstrated the first secondary structure measurements of pre-mRNAs in vivo, obtained with a high-throughput nuclear RNA structure probing method. Michael Clark (University of Oxford, UK) outlined a novel technique combining full-length cDNA sequencing and targeted RNA sequencing, which aims to assist isoform quantification. Stefanie Ebersberger (Institute of Molecular Biology, Mainz, Germany) presented an in vitro derivate of individual-nucleotide resolution CLIP (iCLIP) for generating the intrinsic binding landscape of an RBP, and the computational tools for comparison with iCLIP.

## Conclusions and outlook

The wealth of material presented in the talks and posters naturally stimulated lively discussions about the outstanding challenges in the field. We try here to capture the spirit of these discussions, naturally from our own (biased) perspective. One major impression is that, while many of the data-generating technologies are increasingly becoming quantitative, many of the interpretations still rely on qualitative models, rather than predictive mathematical models. A related topic is the almost complete absence of modeling efforts towards illustrating the dynamical aspects of RNA life. One exception was the talk by Nacho Molina (Institute of Genetics and Molecular and Cellular Biology, Strasbourg, France) on transcriptional control by transcription factors in single cells, which heavily drew on modeling the stochastic dynamics of transcription initiation. Similar models for understanding the post-transcriptional dynamics of RNAs are in short supply and represent a major area of future development. Finally, most computational talks address single data types; most likely, novel biological insights could arise from joint modeling of multiple data types. Integrative models featured in the posters of Philipp Boss (Max Delbrück Center for Molecular Medicine, Berlin) in the context of integrating different CLIP protocols and Ronny Lorenz (University of Vienna), who described generic methods to integrate auxiliary data in structure prediction. We expect that such approaches will become more widespread in the future, and look forward to hearing more about them at the next Computational RNA Biology conference in 2 years time.

## Abbreviations

CAGE: Cap analysis of gene expression
CLIP: Cross-linking and immunoprecipitation
CLASH: Cross-linking, ligation and sequencing of hybrids
CRAC: Cross-linking and cDNA
ENCODE: Encyclopedia of DNA elements
iCLIP: Individual-nucleotide resolution CLIP
lincRNAs: Long intergenic ncRNAs
ncRNA: Non-coding RNA
NGS: Next-generation sequencing
RBPs: RNA-binding proteins
SaTAnn: Splice-aware translational annotation
UV: Ultraviolet

